# Case report: two cases of extremely rare primary pure squamous cell carcinoma of the breast

**DOI:** 10.1097/MD.0000000000012340

**Published:** 2018-09-14

**Authors:** Toshihiko Yoneto, Kenichiro Hasumi, Takayuki Yoshimoto, Nobukazu Takahashi, Yasutaka Takeda

**Affiliations:** a Department of General Medicine, Hijirigaoka Hospital; b Department of Immunoregulation, Institute of Medical Science, Tokyo Medical University; c Department of Surgery, Oguchi-higashi General Hospital; d Breast Oncology Center, Fukujuji Hospital, Tokyo, Japan.

**Keywords:** breast cancer, primary pure squamous cell carcinoma, triple-negative breast cancer

## Abstract

**Rationale:**

Since primary pure squamous cell carcinoma of the breast is a rare disease, few reports describe the characteristic findings on performing preoperative imaging that can be used to distinguish it from normal breast cancer. The rapid evolution and lack of an established method of treatment has resulted in several reports of advanced cases of primary pure squamous cell carcinoma of the breast.

**Patient concerns:**

Case 1 was a 44-year-old woman with an elastic, hard tumor in the left C region. Ultrasonographic analysis revealed a maximal 11-mm hypoechoic area. Histologically, the tumor was a well-differentiated squamous cell carcinoma with prominent keratinization, and there was prominent inflammatory cell infiltration, necrosis, and fibrosis. Case 2 was a 58-year-old woman with an elastic, hard tumor in the left C/D region. Ultrasonographic analysis revealed a maximal 31-mm hypoechoic area with partially calcified areas and a hyperechoic margin. Histologically, the tumor was a squamous cell carcinoma with prominent keratinization exhibiting an infiltrative growth pattern. The tumor had no connection to the epidermis and partially transitioned into the atypical ductal epithelium in the area surrounding the focus.

**Diagnoses:**

The patient in Case 1 was preoperatively diagnosed with T1cN0M0 Stage I cancer of the left breast, but both patients were finally diagnosed with T2N0M0 Stage IIA cancer.

**Interventions:**

Case 1: left partial mastectomy and axillary lymph node dissection were performed. The patient was administered 4 courses of FEC100 and 4 courses of DTX as postoperative adjuvant therapy. Case 2: left modified radical mastectomy and axillary lymph node dissection were performed without any postoperative adjuvant therapy.

**Outcomes:**

Case 1: no sign of relapse was observed, but the patient moved away from the area to another hospital in March 2014 and eventually died due to relapse in January 2016. Case 2: four years after surgery, no relapse has been observed.

**Lessons:**

We should always keep the presence of primary pure squamous cell carcinoma among breast cancers in mind although the crisis rate is very low. Due to its high malignancy, needle biopsy and aspiration biopsy cytology should be performed to make a definitive diagnosis.

## 1 Introduction

Primary pure squamous cell carcinoma of the breast is a relatively rare disease; according to the General Rules for Clinical and Pathological Recording of Breast Cancer published by the Japanese Breast Cancer Society, it comprises only 0.1% to 0.4% of all cases of breast cancer.^[1–3]^ There have been no reports regarding characteristic findings that facilitate a diagnosis of primary squamous cell carcinoma of the breast in contrast to normal breast cancer based on the findings of preoperative imaging. Moreover, it is thought that there are no options but to perform needle biopsy and aspiration biopsy cytology to obtain a definite diagnosis.^[4,5]^ These tumors have a rapid rate of evolution, which is considered to be reflected in the higher rate of lymph node metastasis than normal breast cancer.^[6]^ Indeed, there are several reports of cases that have become advanced due to the lack of an established treatment method.^[4]^ In this paper, we report 2 cases of primary pure squamous cell carcinoma of the breast and provide an extensive review of the literature.

## 2 Case presentation

Approval by an ethics committee or institutional review board was not necessary, because this is a noninvasive follow-up observation study. We obtained verbal informed consents from the patients for this study.

### 2.1 Case 1

A 44-year-old woman with no remarkable family history was admitted to our hospital with the following medical history: after undergoing modified radical mastectomy for cancer of the right breast (papillotubular carcinoma, T3N1M0) in March 2009 at another hospital, the patient received postoperative chemotherapy (4 courses of docetaxel+trastuzumab followed by 14 courses of trastuzumab). However, in March 2011, local excision was performed because of a recurrence that appeared at the site of the surgery, and adjuvant chemotherapy was administered. No relapses were observed thereafter.

When the patient was admitted to our hospital, the patient was of moderate build, was well nourished, had no yellowing of the bulbar conjunctiva, had no conjunctival pallor, and displayed no unusual finding in the heart or lungs. The abdomen was flat; the liver and spleen were not palpable. No remarkable finding was observed. An elastic, hard tumor approximately 20 mm in diameter with relatively distinct boundaries was felt in the left C region. The tumor was observed to be not fixed to the pectoral muscle, and it had not infiltrated the skin. No abnormal nipple discharge was observed, and axillary lymph nodes were not palpable. Peripheral blood and blood biochemistry test results were normal.

**Figure 1:**
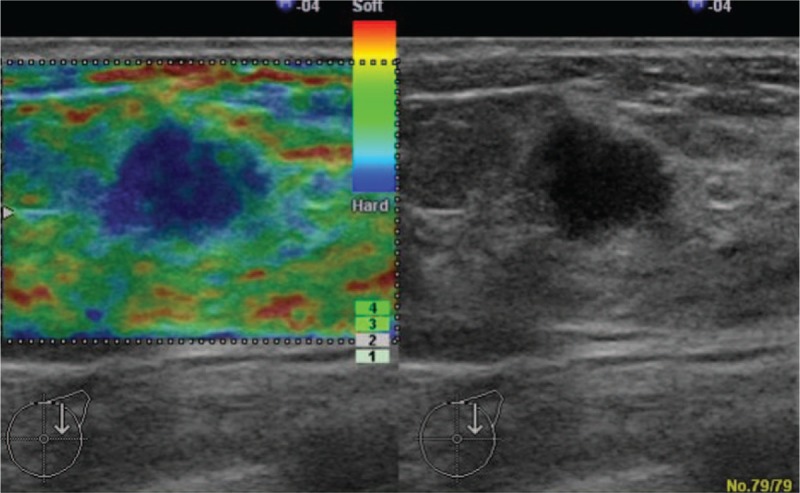
A 10 × 11 × 9 mm hypoechoic area was observed in the C region of the left breast. The lesion had a Tsukuba elasticity score of 2 and internal blood flow, and malignancy was suspected.

**Figure 2:**
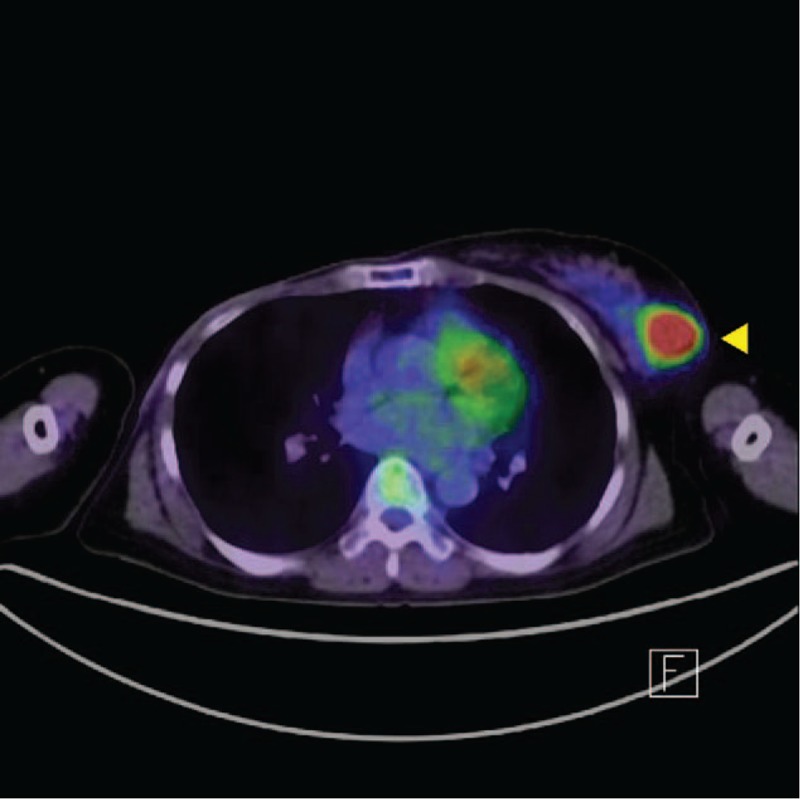
An area of greatly increased uptake was observed in the upper outer quadrant of the left breast (SUV max: 8.2→9.77). A yellow arrowhead in the radiograph indicates the lesion.

By mammographic examination, a high-density tumor with irregular margins and internal calcification was observed and was classified in Category 4. Because the imaging system at our institution has been changed and all previous imaging data were deleted, there are no mammograms to display here. Ultrasonographic analysis revealed that a 10 × 11 × 9 mm hypoechoic area was observed in the C region of the left breast. The lesion had a Tsukuba elasticity score of 2 and internal blood flow, and malignancy was suspected (Fig. 1). By positron emission tomography and X-ray computed tomography (PET-CT), an area of greatly increased uptake was observed in the upper outer quadrant of the left breast (SUV max: 8.2→9.77) (Fig. 2).

Based on the above characteristics, the patient was given a preoperative diagnosis of T1cN0M0 Stage I cancer of the left breast, and left partial mastectomy and axillary lymph node dissection were performed.

**Figure 3:**
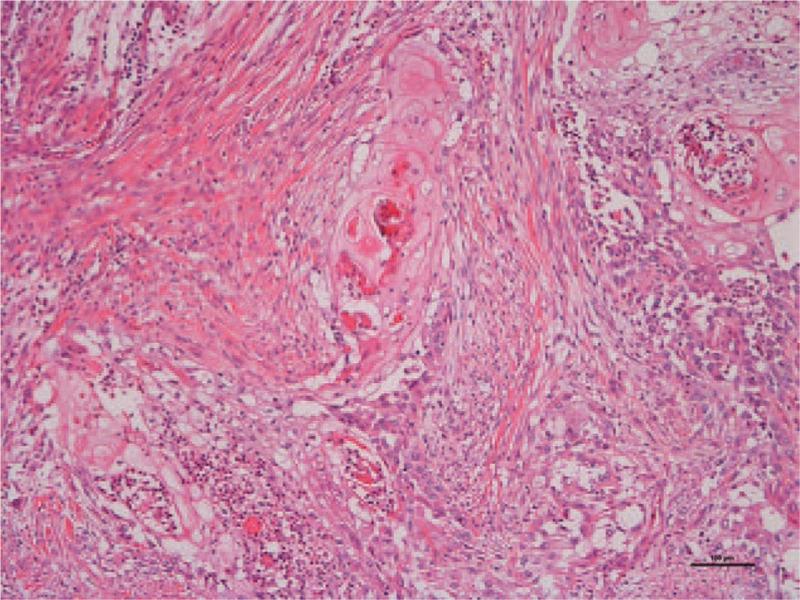
Histological findings with hematoxylin and eosin staining at original magnification ×100. The tumor was a well-differentiated squamous cell carcinoma with prominent keratinization, and there was prominent inflammatory cell infiltration, necrosis, and fibrosis in the surrounding area. The specimen was ly(−), v(−) and did not appear to have any clear sign of vascular invasion. The lymph node was pN0 (0/9):Level I-0/8, Level II-0/1 and contained no metastasis.

The tumor was a solid light gray nodular lesion with distinct borders and measured 27 × 23 × 23 mm. Histologically, the tumor was a well-differentiated squamous cell carcinoma with prominent keratinization, and there was prominent inflammatory cell infiltration, necrosis, and fibrosis. These findings were consistent with primary pure squamous cell carcinoma of the breast. The specimen was ly(−), v(−) and did not appear to have any clear sign of vascular invasion. The lymph node was pN0 (0/9): Level I-0/8, Level II-0/1, and no lymph node metastasis was observed (Fig. 3). Immunohistological staining revealed that hormone receptors were negative, with estrogen receptors (ERs) at ≤1% and progesterone receptors (PgRs) at ≤1%. The HER2 score was 1+ but is only a reference value.

Based on the above findings, the final diagnosis was cancer of the left breast, T2N0M0 Stage IIA.

The postoperative clinical course of the patient was favorable, and the patient was administered 4 courses of FEC100 and 4 courses of DTX as postoperative adjuvant therapy. Observation was continued in the outpatient department, and no sign of relapse was observed. However, the patient moved away from the area to another hospital in March 2014, and eventually died due to relapse in January 2016.

### 2.2 Case 2

A 58-year-old woman with no remarkable medical and family history was admitted to our hospital. When the patient was admitted to our hospital, the patient was of moderate build, was well nourished, had no yellowing of the bulbar conjunctiva, had no conjunctival pallor, and displayed no unusual finding in the heart or lungs. The abdomen was flat; the liver and spleen were not palpable. No remarkable finding was observed. An elastic, hard tumor approximately 30 mm in diameter with relatively distinct boundaries was felt in the left C/D region. The tumor was observed to be not fixed to the pectoral muscle and had not infiltrated the skin. No abnormal nipple discharge was observed, and axillary lymph nodes were not palpable. Peripheral blood and blood biochemistry test results were normal.

**Figure 4:**
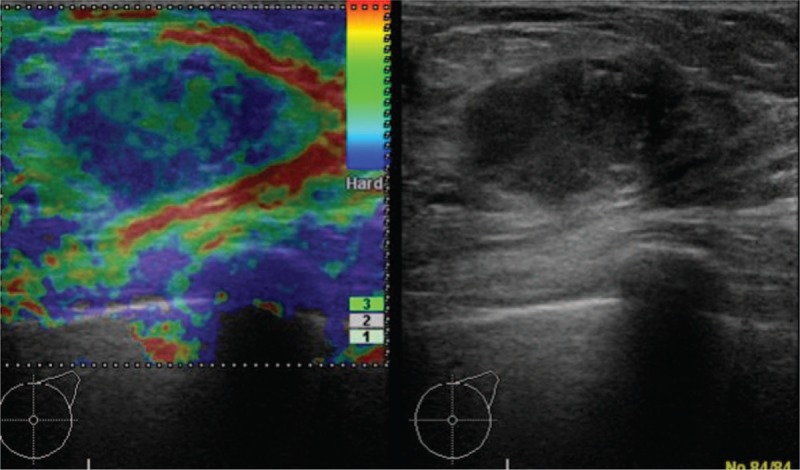
A local asymmetric shadow was observed in the left M area, and it was judged to be Category 3.

**Figure 5:**
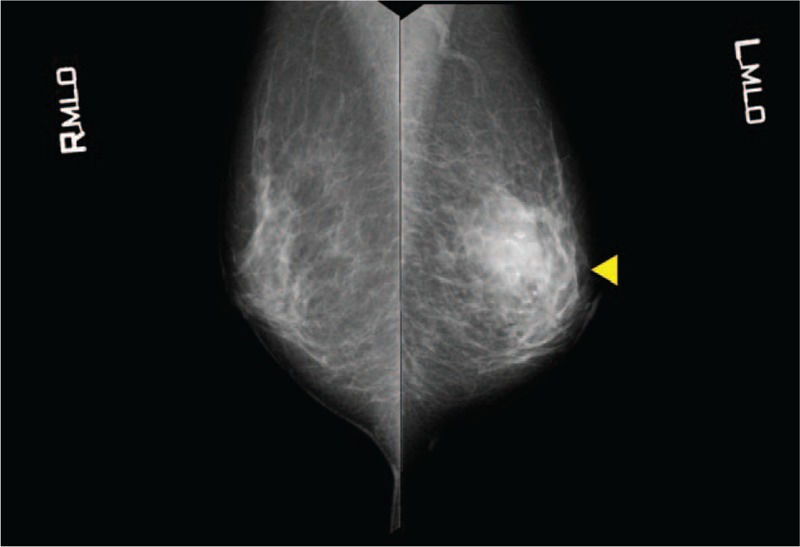
A hypoechoic area measuring 18 × 31 × 24 mm with partially calcified areas and a hyperechoic margin was observed in the C/D region of the left breast. The lesion had a Tsukuba elasticity score of 2 and internal blood flow and strongly suggested malignancy. A yellow arrowhead in the radiograph indicates the lesion.

By mammographic examination, a local asymmetric shadow was observed in the left M area, and the tumor was classified in Category 3 (Fig. 4). Ultrasonographic analysis revealed that a hypoechoic area measuring 18 × 31 × 24 mm with partially calcified areas and a hyperechoic margin was observed in the C/D region of the left breast. The lesion had a Tsukuba elasticity score of 2 and internal blood flow, strongly suggesting malignancy (Fig. 5). By CT examination, a thoracoabdominal CT scan did not reveal anything unusual, such as enlarged axillary lymph nodes or distant metastases in other organs.

Based on the above findings, the patient was given a preoperative diagnosis of T2N0M0 Stage IIA cancer of the left breast, and left modified radical mastectomy and axillary lymph node dissection were performed.

**Figure 6:**
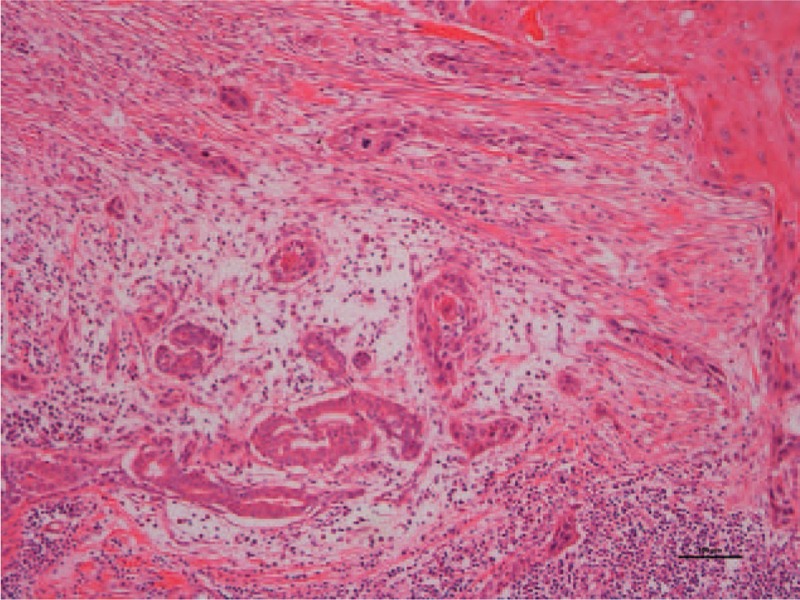
Histological findings with hematoxylin and eosin staining at original magnification ×100. The tumor was a squamous cell carcinoma with prominent keratinization that exhibited an infiltrative growth pattern. The tumor had no connection to the epidermis and partly transitioned into the atypical ductal epithelium (atypical metaplasia) in the area surrounding the focus, which was consistent with primary breast cancer.

The tumor was a solid, light gray nodular lesion with distinct borders and measured 32×22 mm. Histologically, the tumor was a squamous cell carcinoma with prominent keratinization exhibiting an infiltrative growth pattern. The tumor had no connection to the epidermis and partially transitioned into the atypical ductal epithelium (atypical metaplasia) in the area surrounding the focus. These findings were consistent with primary pure squamous cell carcinoma of the breast. The resection margin tested negative. The specimen was ly(−), v(−) and did not appear to have any clear sign of vascular invasion. The lymph node was pN0 (0/20): Level I-0/18, Sentinel-0/2, and there was no lymph node metastasis (Fig. 6). Immunohistological staining revealed that hormone receptors were negative, with ERs at ≤1% and PgRs at ≤1% (both tested positive). The HER2 score was 1+, but it is only a reference value.

Based on the above observations, the final diagnosis was cancer of the left breast, T2N0M0 Stage IIA.

As skin necrosis was observed at the site of surgery, debridement was performed to control necrosis. As the patient did not wish to receive postoperative adjuvant therapy, she was only observed. The patient is periodically examined using full-body or local diagnostic imaging in the outpatient department and currently, 4 years after surgery, no relapse has been observed.

## 3 Discussion

Primary pure squamous cell carcinoma of the breast was first reported in 1936 by Pasternack and Wirth.^[1]^ It is a relatively rare histological type of breast cancer that comprises only 0.1% to 0.4% of all cases of breast cancer.^[2,3]^ It is defined as a special type of cancer accompanied by squamous metaplasia, and it displays both stratification as well as keratinization and/or intercellular bridges.^[7]^ These carcinomas are classified based on the presence or absence of an adenocarcinoma component as either mixed or pure carcinomas, respectively. Cases displaying only a pure squamous cell carcinoma component are extremely rare and comprise 0.046% to 0.28% of all cases of breast cancer.^[8]^ In the present report, both cases were considered to be of the pure type.

It has been reported that these cancers occur at a slightly more advanced age than that at which normal breast cancers appear; however, there are other reports stating that age at onset is the same.^[4,9]^ Moreover, studies have reported that tumor diameters are somewhat larger than other histological types^[4,9]^ and that they are often accompanied by necrotic foci, hemorrhagic foci, cyst formation, and inflammatory changes. This is because the tumors have a rapid rate of evolution, and therefore the frequency of lymph node metastases is relatively higher than that of normal breast cancer,^[6]^ although there is also a report showing that the frequency is low.^[10]^ In the present cases, the tumor from Case 1 was 27 mm in diameter and that from Case 2 was 32 mm in diameter; although both tumors were classified as T2, they cannot be called extremely large tumors, and lymph node metastases were not observed in either case. Moreover, both cases tested negative for ERs and PgRs. It is generally considered that squamous cell cancers of the breast frequently test negative for hormone receptors.^[11]^ Some reports have discussed how this is connected with EGFR and HER2, and suggested that EGFR and HER2 are generally considered to be important prognostic factors for breast cancer and are negatively correlated with ER.^[12]^ It has been reported that preoperative mammograms often show tumor shadows, but spicula formation and calcification are not common.^[13]^ Consistent with this report, the present cases exhibited tumor shadows, but they did not clearly display any spicula. As cystic and tumor shadows are commonly observed in breast ultrasound tests, both cases showed tumor shadows with internal blood flow and some of the findings indicated malignancy. As there have been no reports of preoperative imaging findings that can be used to distinguish between primary squamous cell carcinoma of the breast and normal breast cancer, it is thought that the only way to make a definite diagnosis is to perform needle biopsy and aspiration biopsy cytology.^[4,5]^ It is relatively easy to diagnose breast cancer as primary when adenocarcinoma cells are present together with squamous cell carcinoma. However, in the case of breast cancer with only a pure squamous cell carcinoma component, it is important to discriminate it from metastatic breast cancer.^[5]^

As only a small proportion of cases are hormone-positive, most do not require endocrine therapy.^[6]^ In contrast, another report indicates that treatment similar to that used for normal breast cancer should be selected.^[9]^ Chemotherapy is often chosen for lymph node-positive cases as for other kinds of breast cancer. However, efficacy of drugs such as TS-1, CDDP, and eribulin used for other histological types of breast cancer is poor.^[13,14]^ Because there is currently no established chemotherapy, multidisciplinary clinical trials are eagerly awaited.

Collectively, we have thus experienced 2 cases of extremely rare primary pure squamous cell carcinoma of the breast Stage IIA, but with quite distinct consequences. As with common histological types of breast cancer, cases that test negative for lymph node metastasis are considered to have a good prognosis.^[15]^ The present cases tested negative for lymph node metastasis, and thus it is thought that they would be associated with a favorable prognosis. However, 1 patient died due to relapse, and we could not make a diagnosis based on preoperative imaging findings between primary squamous cell carcinoma and normal breast cancer. We should therefore always keep the presence of primary pure squamous cell carcinoma in mind although the crisis rate is very low, and perform needle biopsy and aspiration biopsy cytology to make a definitive diagnosis if necessary. Primary pure squamous cell carcinoma has a rapid rate of growth and evolution, and there is no established treatment method, so urgent treatments such as chemotherapy are necessary. Currently, there are no reports concerning other determining factors for prognosis or diagnosis, and we look forward to the publication of additional case reports and future research into the basic pathology of the disease.

## Acknowledgment

The authors express their thanks to Yasuhiro Ishino of the Hijirigaoka Hospital Testing Department for processing the images. No assistance was received from any corporation in relation to this report.

## Author contributions

**Data curation:** Toshihiko Yoneto.

**Formal analysis:** Toshihiko Yoneto.

**Resources:** Yasutaka Takeda.

**Writing – original draft:** Toshihiko Yoneto.

**Writing – review & editing:** Toshihiko Yoneto, Kenichiro Hasumi, Takayuki Yoshimoto, Nobukazu Takahashi, Yasutaka Takeda.
